# Long-Term Outcomes of Stereotactic Body Radiotherapy (SBRT) for Intraprostatic Relapse after Definitive Radiotherapy for Prostate Cancer: Patterns of Failure and Association between Volume of Irradiation and Late Toxicity

**DOI:** 10.3390/cancers15041180

**Published:** 2023-02-13

**Authors:** Marcin Miszczyk, Małgorzata Kraszkiewicz, Matthias Moll, Konrad Kaminiów, Szymon Sobel, Łukasz Dolla, Piotr Wojcieszek, Paweł Rajwa, Takafumi Yanagisawa, Zuzanna Nowicka, Shahrokh F. Shariat, Gregor Goldner, Leszek Miszczyk, Wojciech Majewski

**Affiliations:** 1 IIIrd Radiotherapy and Chemotherapy Department, Maria Skłodowska-Curie National Research Institute of Oncology, Wybrzeże Armii Krajowej 15, 44-102 Gliwice, Poland; 2 Department of Radiation Oncology, Comprehensive Cancer Center, Medical University of Vienna, Währinger Gürtel 18-20, 1090 Vienna, Austria; 3 Radiotherapy Department, Maria Skłodowska-Curie National Research Institute of Oncology, Wybrzeże Armii Krajowej 15, 44-102 Gliwice, Poland; 4 Radiotherapy Department, Centre Antoine-Lacassagne, 33, Avenue Valombrose, CEDEX2, 06189 Nice, France; 5 Radiotherapy Planning Department, Maria Skłodowska-Curie National Research Institute of Oncology, Wybrzeże Armii Krajowej 15, 44-102 Gliwice, Poland; 6 Brachytherapy Department, Maria Skłodowska-Curie National Research Institute of Oncology, Wybrzeże Armii Krajowej 15, 44-102 Gliwice, Poland; 7 Department of Urology, Comprehensive Cancer Center, Medical University of Vienna, 1090 Vienna, Austria; 8 Department of Urology, Medical University of Silesia, 40-752 Zabrze, Poland; 9 Department of Urology, The Jikei University School of Medicine, Tokyo 105-8461, Japan; 10 Department of Biostatistics and Translational Medicine, Medical University of Łódź, 90-419 Łódź, Poland; 11 Institute for Urology and Reproductive Health, Sechenov University, 119435 Moscow, Russia; 12 Hourani Center for Applied Scientific Research, Al-Ahliyya Amman University, Amman 19328, Jordan; 13 Department of Urology, University of Texas Southwestern Medical Center, Dallas, TX 75230, USA; 14 Department of Urology, Second Faculty of Medicine, Charles University, 12108 Prague, Czech Republic; 15 Department of Urology, Weill Cornell Medical College, New York, NY 10065, USA; 16 Karl Landsteiner Institute of Urology and Andrology, 1090 Vienna, Austria

**Keywords:** prostate cancer, stereotactic body radiotherapy, SBRT, salvage stereotactic body radiotherapy, sSBRT, salvage treatment, long-term outcomes, toxicity

## Abstract

**Simple Summary:**

Despite advancements in prostate cancer treatment, local recurrence remains among the most common patterns of failure. A recent meta-analysis has shown that, in patients initially treated with radiotherapy, salvage re-irradiation seems to be associated with the most favorable toxicity outcomes, albeit based on a limited set of data. This includes high-dose-rate or low-dose-rate brachytherapy and salvage stereotactic body radiotherapy. In this retrospective study, we found that salvage stereotactic body radiotherapy for local recur-rence of prostate cancer after definitive radiotherapy presents a significant risk of grade ≥ 3 adverse effects associated with the volume of re-irradiation..

**Abstract:**

The aim of this retrospective study was to assess the adverse effects and outcomes of salvage re-irradiation with stereotactic body radiotherapy (sSBRT) for local recurrence of prostate cancer (PCa) after definitive radiotherapy (RT). The study was focused on the adverse effects and prognostic factors for treatment toxicity, followed by an analysis of patterns of failure and survival. Patients treated with sSBRT between 2012 and 2020 at a tertiary institution were included. The exclusion criteria were a primary or salvage radical prostatectomy or a palliative sSBRT dose. Patients with oligorecurrence were eligible if all metastatic lesions were treated locally with curative intent. The Kaplan–Meier method was used to estimate time to grade ≥ 3 toxicity, local control (LC), freedom from distant metastases (FFDM), progression-free survival (PFS), biochemical control (BC) and overall survival (OS). The differences between groups (focal vs. whole-gland sSBRT) were compared using the log-rank test. The Cox proportional hazards model was used to assess prognostic factors for the listed endpoints. A total of 56 patients with a median age of 70.9 years and a median follow-up of 38.6 months were included in the analysis. The majority of them received local sSBRT only (45; 80.4%), while the rest were simultaneously treated for oligometastases (11; 19.6%). Overall, 18 (32.1%) patients experienced any grade ≥ 3 toxicity, including 1 (6.7%) patient who received focal sSBRT, and 17 (41.5%) patients treated with whole-gland sSBRT. The Planning Target Volume (per cc; HR 1.01; 95% CI 1–1.02; *p* = 0.025) and use of ADT (yes vs. no; HR 0.35; 95%CI 0.13–0.93; *p* = 0.035) were independent prognostic factors for the risk of grade ≥ 3 toxicity. The estimated rate of grade ≥ 3 adverse events was significantly higher (43.8% vs. 7.1% at 2 years; *p* = 0.006), and there was no improvement in the LC (92.9% vs. 85.3% at 2 years; *p* = 0.759) in patients treated with whole-gland sSBRT compared to focal sSBRT. The 2- and 5-year LC were 87.6% and 47.9%, respectively; the 2- and 5-year FFDM were 72.7% and 42.8%, respectively; and the 2- and 5-year PFS were 67.9% and 28.7%, respectively. The primary pattern of failure was distant metastasis. The sSBRT for local recurrence of PCa after definitive RT was associated with a high risk of severe grade ≥ 3 toxicity, which significantly increased with the volume and extent of re-irradiation.

## 1. Introduction

Despite advancements in tumor control through dose escalation [1,2,3], up to 8–15% of patients treated with modern radiotherapy (RT) for prostate cancer (PCa) experience biochemical recurrence in the first five years after treatment [4]. The introduction of focally dose-escalated radiotherapy schemes might significantly reduce intraprostatic clinical failures [5], but the prostate and seminal vesicles remain the most common first-recurrence sites after conventional radiotherapy, with a 3.5–14.6% cumulative 8-year incidence [6]. To avoid androgen-deprivation therapy (ADT), which is associated with adverse effects and an impact on the quality of life [7,8], local therapeutic strategies such as salvage treatment have been implemented to delay progression or even cure the disease. These modalities include salvage radical prostatectomy, RT, brachytherapy (BT), cryotherapy and high-intensity focused ultrasound (HIFU). A recent meta-analysis has shown that oncological outcomes are similar between methods, but the rate of severe genito-urinary (GU) and gastrointestinal (GI) toxicity favors re-irradiation [9]. Salvage stereotactic body radiotherapy (sSBRT) presented the lowest rate of severe GU adverse effects and an acceptable GI toxicity profile; however, the estimations were based on a significantly smaller study with a much shorter follow-up. The promising initial outcomes led to the creation of the first consensus guidelines on sSBRT and a wider acceptance of this treatment modality [10].

Due to the rising concern associated with clinical observations of significant late toxicity, we present data with a long follow-up focused on treatment safety and patterns of failure. To the best of our knowledge, we are the first to present a significant association between the volume of re-irradiation and the risk of late toxicity in patients treated with sSBRT for locally recurrent PCa following definitive RT.

## 2. Materials and Methods

This retrospective study included patients treated with hypofractionated sSBRT for local recurrence of PCa after definitive RT at a tertiary institution between 2012 and 2020. The exclusion criteria were radical prostatectomy as primary or salvage treatment or palliative doses of sSBRT (defined as Biologically Effective Dose [BED] <100 Gy). No prior local salvage treatment to the prostate before sSBRT was allowed. Concomitant oligometastases (n ≤ 5) were not considered exclusion criteria if sSBRT was combined with metastases-directed therapy (intention to treat). In each applicable case, the treatment consisted of high-dose stereotactic radiotherapy delivered to each of the metastatic lesions with curative intent. The most common treatment schedule was 36.25 Gy in five fractions delivered using a CyberKnife^TM^ linear accelerator to the Planning Target Volume (PTV) defined as the whole prostate with a 5 mm margin in each direction except for 3 mm margin posteriorly. The use of ADT was permitted and, in the majority of applicable cases, prescribed at the discretion of the attending urologist. The detailed study group description is presented in Table 1.

The starting point for the analysis of complications and oncologic outcomes was the date of the first sSBRT fraction. Local control (LC) was defined as time to local failure (based on radiological findings). Freedom from distant metastases (FFDM) was defined as the time to the occurrence of distant metastases. Biochemical control (BC) was calculated as time to biochemical failure defined according to the Phoenix criterion (nadir + 2 ng/mL). In cases where the endpoint did not occur, LC, FFDM and BC were censored with the date of the last clinical FU. Overall survival (OS) was defined as time to death and otherwise censored with the last known data point at which the patient was alive (based on census data). Distant metastases and death were endpoints for metastases-free survival (MFS). Any events involving local failure, distant metastases or death were endpoints for progression-free survival (PFS). Both MFS and PFS were censored similarly to OS. In the case of patients treated simultaneously for local recurrence and oligometastases, the diagnosis of new lesions or radiologic progression of previously treated metastases based on the RECIST criteria (version 1.1) [11] was considered as ‘distant metastases’ endpoint for MFS and PFS. In patients with simultaneous distant and local failure, distant metastases were reported as 1st PFS event.

The toxicity was graded according to the Common Terminology Criteria for Adverse Events (CTCAE), version 5.0 [12]. Severe toxicity was defined as grade ≥ 3 toxicity. Kaplan–Meier curves and Cox regression models were calculated using the time from the start of sSBRT to the occurrence of the first grade ≥ 3 toxicity (complete observations) or end of clinical FU (censored observations). The serious adverse events (SAE) label was used according to the Food and Drug Administration (FDA) definition [13].

The follow-up was based on institutional patients’ medical records. Regardless of routine medical history, patients were contacted to perform a study-specific control visit with a focus on adverse effects. In the case of deceased patients, previously approved representatives were contacted for the possibility of sharing the medical history. The patients/representatives were informed that the study-specific control visit was not obligatory and its primary aim was research-oriented. Whenever possible, they were scheduled on the same day as their next routine visit to the hospital.

The statistical analysis included the Kaplan–Meier method for estimation of survival and occurrence of toxicity and the log-rank test for assessment of differences between groups (focal sSBRT vs. whole-gland sSBRT). Cox proportional hazards model was used for the estimation of prognostic factors for clinical endpoints and toxicity (including time to occurrence of grade ≥ 3 toxicity). Variables at a *p*-level of <0.15 were selected for the multivariate analysis. The correlations between related significant variables in univariate Cox models were assessed with R-Spearman test to avoid co-linearity in the multivariable model. K-nearest neighbors algorithm was used to fill in missing data. Outcomes at a *p*-level of <0.05 were considered to be statistically significant. The statistical analysis was conducted using Statistica 13.3 software by StatSoft (TIBCO Software, Palo Alto, CA, USA).

The study protocol was approved by the bioethical committee of the Maria Sklodowska-Curie National Research Institute of Oncology, No. KB/430-09/22. Given the retrospective nature of this study, informed consent for participating in it was not deemed necessary. The patient’s (or representative’s) consent was necessary for conducting the study-specific visit.

## 3. Results

Between 2012 and 2020, 96 patients were treated for a local recurrence of PCa after RT (external-beam RT, BT or a combination of both) with hypofractionated sSBRT as salvage treatment at our department. Out of the initial cohort, 40 patients did not meet the inclusion and exclusion criteria, primarily due to having received a palliative sSBRT dose or a previous radical prostatectomy. The final study group consisted of 56 patients with a median age of 70.9 years (interquartile range [IQR] 66.9–77.7) and a median follow-up (FU) of 38.6 months (IQR 18.7–53.9). The diagnosis of local recurrence was made using (18)F-fluorocholine-PET (37.5%), PSMA-PET (37.5%), multiparametric MRI (18.8%) or CT in one case (seminal vesicle recurrence). The majority of the patients received local sSBRT only (45; 80.4%), while the remainder were also simultaneously treated with metastases-directed therapy (11; 19.6%) for oligometastases. A detailed description of the study group is presented in Table 1 and Supplementary File S1. The results of the uni- and multivariate analyses for grade ≥ 3 toxicity, LC, FFDM, OS and PFS can be found in Supplementary File S2 (Supplementary Tables S1–S6, respectively). 

### 3.1. Treatment Toxicity

Overall, 18 (32.1%) patients experienced any grade ≥ 3 toxicity, including one (6.7%) patient who received focal sSBRT and 17 (41.5%) patients treated with whole-gland sSBRT. Grade ≥2 toxicity was observed in 35 (62.5%) patients, including 6 (40%) patients who received focal sSBRT and 29 (70.7%) who received whole-gland sSBRT. 

A total of 10 grade 4 adverse events (AEs), 28 grade 3 AEs, 60 grade 2 AEs, and 85 grade 1 AEs were reported, including 35 serious adverse events (SAEs). All AEs and SAEs are described in Supplementary File S1, along with a brief medical history of the patients. The number of patients reporting each AE is presented in Table 2.

In the univariable analysis, the risk of grade 3+ toxicity was significantly associated with the size of the Planning Target Volume (PTV) (per cc; HR 1.01; 95%CI 1–1.03; *p* = 0.013), the extent of sSBRT (focal vs. whole-gland; HR 0.11; 95%CI 0.01–0.83; *p* = 0.032) and ADT (yes vs. no; HR 0.32; 95%CI 0.12–0.84; *p* = 0.02), as shown in Supplementary Table S1. Both PTV (per cc; HR 1.01; 95%CI 1–1.02; *p* = 0.025) and ADT (yes vs. no; HR 0.35; 95%CI 0.13–0.93; *p* = 0.035) remained significant prognostic factors in the multivariable analysis.

**Figure 1:**
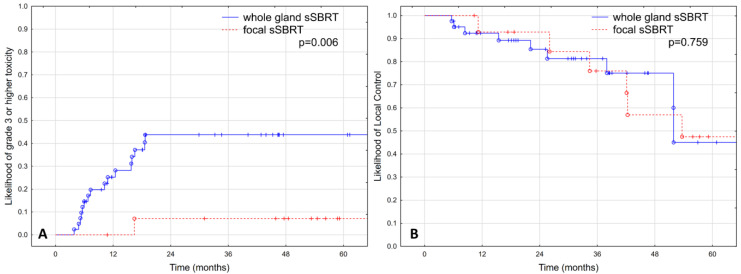
Rate of grade ≥3 adverse events (**A**) and local control (**B**) depending on the extent of irradiation in 56 patients treated with hypofractionated salvage re-irradiation for local post-radiotherapy prostate cancer recurrence.

There was a statistically significant difference in the estimated occurrence of grade ≥ 3 toxicity between patients treated with focal and whole-gland sSBRT in favor of the focal treatment group (43.8% vs. 7.1% at 2 years; *p* = 0.006), as shown in Figure 1A.

### 3.2. Local Control

**Figure 2:**
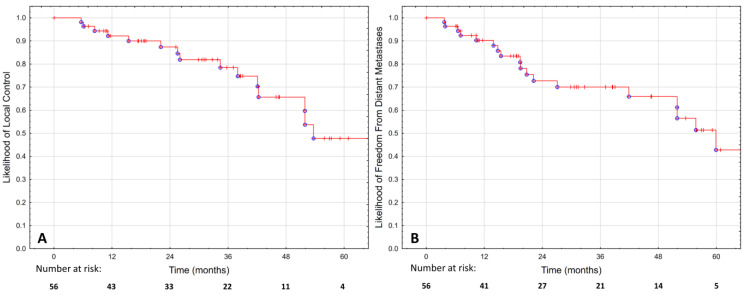
Local control (**A**) and freedom from distant metastases (**B**) in 56 patients treated with hypofractionated salvage re-irradiation for local post-radiotherapy prostate cancer recurrence.

The estimated LC was 87.6% at two years and 47.9% at five years, as shown in Figure 2A. No significant difference was observed in LC between patients treated with focal and whole-gland sSBRT, as shown in Figure 1B (*p* = 0.759).

The local recurrence was predicted by parameters related to the initial radiotherapy. ISUP Grade Group (4–5 vs. 1; HR 4.86; 95%CI 1.06–22.36; *p* = 0.042), TNM T-stage (T2b-c vs. T1c-T2a; HR 11.34; 95%CI 2.43–52.87; *p* = 0.001) and maximum PSA (HR 1.01; 95%CI 1–1.01; *p* = 0.021) at primary treatment remained independent prognostic factors for local failure, as presented in Supplementary Table S2.

### 3.3. Distant Metastases

The FFDM was 72.7% at two years and 42.8% at five years, as shown in Figure 2B. Metastases were most commonly found in the bones (50%), lymph nodes (25%) or both (25%). The diagnosis was made using (18)F-fluorocholine-PET (45%), PSMA-PET (25%), MRI (15%), scintigraphy (10%) or CT in one case. In the majority of cases (55%), there were more than five new metastases. In the remaining 45% of cases, oligo-progression was diagnosed, and 78% of those patients had metastases-directed therapy for all new lesions.

The risk of distant metastases was significantly increased in the multivariable analysis only in patients with a higher initial ISUP Grade Group (ISUP 4–5 vs. 1; HR 5.01; 95%CI 1.27–19.76; *p* = 0.021), as shown in Supplementary Table S3.

### 3.4. Progression-Free Survival 

The PFS was 67.9% at two years and 28.7% at five years. There were no significant differences between patients irradiated with focal and whole-gland sSBRT (*p* = 0.823), as shown in Supplementary File S3. Over the course of FU, PFS events were observed in a total of 36 (64.3%) patients. The most common event was distant metastases (17; 30.4%), followed by death (13; 23.2%) or local recurrence (6; 10.7%).

Consistent with previous findings, the initial ISUP Grade Group (4–5 vs. 1; HR 4.15; 95%CI 1.73–9.94; *p* = 0.001) and TNM T-stage (T2b-c vs. T1c-T2a; HR 2.89; 95%CI 1.13–7.36; *p* = 0.026) at primary irradiation were independent prognostic factors for PFS, as shown in Supplementary Table S4.

### 3.5. Biochemical Control

The estimated BC was 78.4% at two years and 44.9% at five years, with no significant differences between patients irradiated with focal and whole-gland sSBRT (*p* = 0.548), as shown in Supplementary File S3. In the MVA, the risk of biochemical failure was significantly associated only with the initial ISUP Grade Group (4–5 vs. 1; HR 8.85; 95% CI 2.16–36.22; *p* = 0.002), as presented in Supplementary Table S5.

### 3.6. Overall Survival

**Figure 3:**
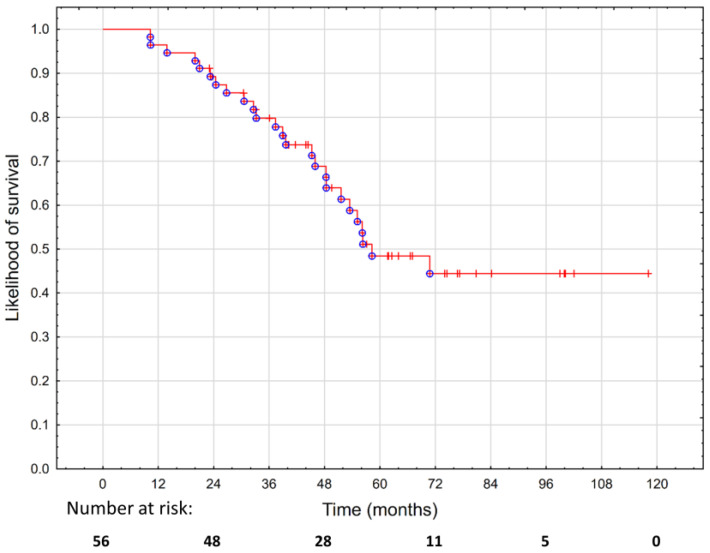
Overall survival in 56 patients treated with hypofractionated salvage re-irradiation for local post-radiotherapy prostate cancer recurrence.

The OS was 89.2% at two years and 48.5% at five years, as shown in Figure 3. We did not find any statistically significant predictors of survival except for the PTV, which was associated with an increased risk of death (*p* = 0.019; HR 1.01; 95%CI 1–1.03), as shown in Supplementary Table S6.

## 4. Discussion

In this article, we present evidence that, over a longer FU period, the rate of serious AEs (grade ≥3) in patients treated with sSBRT can be significantly higher than previously reported. First, we have shown the crucial impact of treatment volume, which was likely associated with the high treatment toxicity in our cohort. Second, we have shown that the primary pattern of failure was distant metastases, including early dissemination, implying that precise patient selection is necessary for the local treatment to be effective. Third, we discovered that variables related to primary treatment correlated significantly with sSBRT outcomes.

The majority of the authors do not differentiate between sSBRT following definitive RT and sSBRT after adjuvant RT following radical prostatectomy. Based on our experience, the latter group receives a lower RT dose at primary treatment, and the sSBRT is delivered to a lower volume due to a more precise identification of the lesion in the postoperative setting in the absence of the prostate gland. Our findings suggest that a ‘low-volume’ approach should be adapted for all sSBRT. The only grade ≥3 AE case in the focal sSBRT group was pelvic soft tissue necrosis following surgical intervention for local recurrence. There was another case of necrosis following surgery in our cohort resulting from rectal cancer. We believe that, in both cases, the possible association with sSBRT should not be disregarded, as the necrosis occurred in the previously re-irradiated region.

Over 73% of the patients in our study group and 61% of the patients included in the MASTER meta-analysis were treated with whole-prostate re-irradiation [9]. Based on our findings, focal sSBRT is superior in terms of toxicity and equivalent in efficacy, which is consistent with the findings of a meta-analysis by Corkum et al. that showed both increased toxicity and no improvement in oncological outcomes of whole-prostate sSBRT [14]. The favorable toxicity and efficacy profile shown in the meta-analysis was based on data with a relatively low median FU of 26 months. In the two largest studies included in the meta-analysis, Fuller et al. presented data on 50 patients treated with whole-prostate sSBRT in a prospective trial [15] and a median FU of 44 months. The authors reported only an 8% rate of grade 3+ adverse effects at 5 years and a 60% 5-year biochemical PFS. However, despite using the whole-gland approach, the authors employed a ‘0 mm margin for PTV’, resulting in irradiated volumes that were approximately three times smaller than those of our patients. Pasquier et al. reported on 100 patients with a median FU of 29.3 months, showing a 3-year biochemical recurrence-free survival of 55% and virtually no grade ≥ 3 toxicity [16]. The median PTV was approximately twice as low as in our study. Finally, a partial-prostate sSBRT study reported only two cases of grade ≥3 AEs at a median FU of 25.4 months in 44 patients. The median PTV was more than twice as low as in our study, which seems to confirm the importance of treatment volume reduction. The only published series with a long follow-up and a high prevalence of adverse effects investigated conventional re-irradiation based on older radiotherapy techniques, presumably resulting in larger irradiated volumes [17]. Other authors have proposed several possible solutions for increasing the conformity of sSBRT. While there is a consensus on the pivotal value of MR for target definition [10], MR can also be used for image verification and daily adaptive radiotherapy to further reduce the dose to organs at risk [18].

There are several established methods for treating localized prostate cancer recurrence after radiotherapy that are associated with relatively similar 2-year (54–81%) and 5-year (50–60%) relapse-free survival (RFS) [9]. In patients with low co-morbidity, life expectancy of >10 years, PSA <10 ng/mL at recurrence, initial ISUP grade 1–3 and initial T1-2 N0 M0 clinical stage, salvage radical prostatectomy may be considered [19,20]. The treatment is associated with a significantly higher risk of complications compared to primary radical prostatectomy, including a higher risk of anastomotic stricture, urinary retention, urinary fistula, abscess and rectal injury [21]. Grade ≥3 AEs mainly occur in the GU domain (21%; 95% CI 16–27), and are less commonly related to GI toxicity (1.9%l 95% CI 0.6–3.7). The cryoablation of the prostate was developed as a potentially equally efficient but safer alternative. Despite the fact that its efficacy seems to be comparable to that of salvage radical prostatectomy, cryoablation has been found to be associated with a similar risk of grade ≥3 GU AEs (15%; 95% CI 10–22) and a minor risk of severe GI AEs (1.7%; 95% CI 1–2.7). Due to the lack of strong evidence and high uncertainty with regard to long-term outcomes, cryotherapy is currently discouraged in routine clinical practice [20]. Thermal ablation can also be performed using high-intensity focused ultrasound (HIFU), but it has low 2- and 5-year RFS rates and a high rate of severe GU toxicity (23%; 95% CI 17–30%) [9]. Similar to cryoablation, HIFU should not be used as a salvage treatment for local recurrences outside of prospective trials [20]. Finally, salvage re-irradiation can be carried out either through BT or external-beam RT. Salvage BT can be performed using the high-dose-rate (HDR) or low-dose-rate (LDR) approach, both yielding similar clinical results and a favorable toxicity profile with only 9.6% and 9.1% risk of severe GU toxicity, and 0% and 2.1% risk of severe GI toxicity, respectively [9].

sSBRT has been reported to be associated with an even lower risk of severe GU (5.6%; 95% CI 1.4–12) and GI (0%; 95% CI 0–1.2) toxicity. However, these estimations were based on significantly smaller study groups and much shorter follow-up periods compared to salvage BT. Our study has shown that treatment toxicity can be considerably higher than previously estimated and significantly associated with the volume of irradiation. Despite a high 2-year PFS of 67.9% in our study, the 5-year PFS was only 28.7%, significantly lower than the 56% 5-year RFS estimated by Valle et al. [9]. It is likely due to the inclusion of CRPC and oligometastatic patients. The high rate of significant treatment toxicity remains a major clinical issue, and, especially in light of its limited efficacy, we believe that, currently, sSBRT should only be considered in selected patients at high-reference centers, preferably in clinical trials or well-documented prospective cohorts.

Our study group’s main failure patterns were distant metastases and a significant subset of patients experiencing local recurrence, both of which were predicted by unfavorable cancer pathology at primary treatment. A similar observation was made in a recent prospective trial on sSBRT and HDR BT. The authors observed a subset of patients with early progression after salvage treatment, mainly represented by a high initial ISUP Grade Group [22]. A Gleason score of >7 predicted worse biochemical control in another study [23]. The model could be improved through the inclusion of the pre-sSBRT ISUP Grade Group. However, these data were not available in one-fourth of the cases (no biopsy, diagnosis based on medical imaging) and, in half of the remaining cases, the histopathological examination was described as ‘adenocarcinoma, unable to determine Gleason grade due to concomitant radiotherapy-induced changes’. Although not significant in our analysis, a recent study suggested that CRPC is associated with a higher risk of distant metastases, which limits the benefit of local salvage [24]. The introduction of routine PSMA-PET-aided focal therapy could improve both the conformity of treatment and the detection of otherwise subclinical metastases [25].

There are several limitations to our study, including the fact that the data were gathered retrospectively and the majority of patients were treated with high-dose sSBRT to the whole prostate. Additionally, despite treatment planning MRIs being used mainly for OAR and whole-gland contouring, diagnostic-grade contrast-enhanced multiparametric MRI for precise lesion delineation was available in only one-third of the cases. The data are inhomogeneous and includes patients with oligometastatic disease and CRPC at salvage, which made it difficult to assess the timing of ADT as many of these patients started hormone treatment significantly earlier than sSBRT. Finally, due to the relatively small and inhomogeneous study group, this study is limited in terms of the assessment of prognostic factors for clinical outcomes. Nevertheless, we believe that our findings are important for the development of sSBRT and show a significant association between the volume of irradiation and the risk of grade ≥ 3 toxicity, which, to the best of our knowledge, has so far been neglected in the literature.

## 5. Conclusions

Salvage whole-gland SBRT for post-radiotherapy prostate cancer recurrence is associated with a significant, volume-dependent risk of grade ≥ 3 toxicity, including a high risk of permanent late toxicity such as fistula or soft tissue necrosis. The introduction of precise image-guided focal treatment and the reduction in treatment margins are necessary for patient safety. 

Despite the relatively high rate of local recurrences, the primary pattern of failure is distant metastases. The risk of progression is associated with the clinical characteristics at primary treatment, including TNM T-stage and ISUP Grade Group.

## Figures and Tables

**Table 1:** Study group description of 56 patients treated with hypofractionated salvage re-irradiation for local post-radiotherapy prostate cancer recurrence.

	Whole Group	Whole-Gland sSBRT	Focal sSBRT
Variable	N = 56	N = 41	N = 15
Age [years]	70.9 (66.9–77.7)	71.5 (66.7–77.8)	70.3 (67.5–77.6)
Follow-up [months]	38.6 (18.7–53.9)	34.3 (18.1–45.3)	54.5 (45.9–71.6)
Primary treatment:			
PSA max [ng/mL]	13.1 (7.39–21.19)	14.3 (8–25)	8.3 (6–16.8)
ISUP Grade Group:			
1	32 (57.1%)	19 (46.3%)	13 (86.7%)
2	9 (16.1%)	8 (19.5%)	1 (6.7%)
3	1 (1.8%)	1 (2.4%)	0 (0%)
4	7 (12.5%)	7 (17.1%)	0 (0%)
5	7 (12.5%)	6 (14.6%)	1 (6.7%)
T stage			
T1c	34 (60.7%)	26 (63.4%)	8 (53.3%)
T2a	3 (5.4%)	2 (4.9%)	1 (6.7%)
T2b	3 (5.4%)	1 (2.4%)	2 (13.3%)
T2c	7 (12.5%)	3 (7.3%)	4 (26.7%)
T3a	1 (1.8%)	1 (2.4%)	0 (0%)
T3b	8 (14.3%)	8 (19.5%)	0 (0%)
N0	98.2%	97.6%	100%
M0	100%	100%	100%
ADT (primary treatment) ^	34 (60.7%)	26 (63.4%)	8 (53.3%)
RT modality:			
EBRT	47 (83.9%)	35 (85.4%)	12 (80%)
BT boost	4 (7.1%)	2 (4.9%)	2 (13.3%)
LDR BT	3 (5.4%)	3 (7.3%)	0 (0%)
HDR BT	2 (3.6%)	1 (2.4%)	1 (6.7%)
PSA nadir [ng/mL]	0.21 (0.03–0.51)	0.2 (0.03–0.51)	0.22 (0.02–0.6)
Salvage treatment:			
Time to salvage [months]	87.5 (60.3–124.5)	87.7 (61.4–115)	80.1 (56.7–132.9)
ADT (salvage treatment) ^	41 (73.2%)	31 (75.6%)	10 (66.7%)
Duration of ADT [months] ^#^	24 (12–54)	24 (6–54)	27 (12–84)
CRPC	13 (23.2%)	9 (22%)	4 (26.7%)
Oligometastatic	11 (19.6%)	8 (19.5%)	3 (20%)
PSA max at salvage [ng/mL]	4.13 (2.59–7.03)	4.11 (2.8–7.05)	4.16 (2.37–5.54)
Biopsy-proven	42 (75%)	34 (82.9%)	8 (53.3%)
Pre-treatment workup:			
(18)F-fluorocholine-PET	34 (60.7%)	25 (61%)	9 (60%)
PSMA-PET	10 (17.9%)	9 (22%)	1 (6.7%)
MRI	19 (33.9%)	13 (31.7%)	6 (40.0%)
GTV/CTV [cc]	32 (14.7–41.4)	34.1 (27.8–41.6)	6.8 (3.3–12.2)
PTV [cc]	66.5 (32.7–79.9)	73.2 (62.4–84.6)	20.6 (10.2–30.6)
Fractionation schedule:			
36.25/7.25 Gy	36 (64.3%)	28 (68.3%)	8 (53.3%)
35/7 Gy	2 (3.6%)	2 (4.9%)	0 (0%)
33.75/6.75 Gy	4 (7.1%)	4 (9.8%)	0 (0%)
30/10 Gy SIB *	4 (7.1%)	1 (2.4%)	3 (20%)
30/6 Gy	3 (5.4%)	1 (2.4%)	2 (13.3%)
30/5 Gy	1 (1.8%)	1 (2.4%)	0 (0%)
27.5/5.5 Gy	4 (7.1%)	3 (7.3%)	1 (6.6%)
22.5/7.5 Gy	1 (1.8%)	1 (2.4%)	0 (0%)
20/10 Gy	1 (1.8%)	0 (0%)	1 (6.6%)

^ patients receiving androgen-deprivation therapy (ADT) as a part of primary or salvage treatment; ^#^ in patients receiving ADT; * Three patients received 30/10 Gy as focal therapy (BED_1.5_ = 230 Gy), and 15/5 Gy to the whole gland (BED_1.5_ = 65 Gy). One patient received two 10 Gy fractions to the whole prostate (BED_1.5_ = 153.3), and a third 10 Gy fraction to the focal lesion (BED_1.5_ = 230 Gy), which was designated as whole-gland irradiation since the dose to the whole gland was above the assumed threshold for radical treatment (BED_1.5_ > 100 Gy).

**Table 2:** Overall occurrence of adverse effects in 56 patients treated with hypofractionated salvage re-irradiation for local post-radiotherapy prostate cancer recurrence.

Adverse Effect:	Total:	%	Grade I	Grade II	Grade III	Grade IV	SAE	%
Genito-urinary toxicity								
Cystitis noninfective	35	62.5%	21	13	1	0	1	1.8%
Urinary frequency	14	25.0%	10	4	N/A	N/A	0	0.0%
Urinary tract obstruction	12	21.4%	9	2	1	0	1	1.8%
Hematuria	8	14.3%	2	4	2	0	2	3.6%
Urinary fistula	7	12.5%	N/A	0	4	3	7	12.5%
Urinary tract infection	6	10.7%	N/A	4	2	0	2	3.6%
Urinary incontinence	6	10.7%	2	3	1	N/A	0	0.0%
Urinary retention	4	7.1%	1	2	1	0	1	1.8%
Urinary urgency	4	7.1%	4	0	N/A	N/A	0	0.0%
Cystitis infective	1	1.8%	0	1	0	0	0	0.0%
Urinary tract pain	1	1.8%	1	0	0	N/A	0	0.0%
Gastro-intestinal toxicity								
Rectal hemorrhage	16	28.6%	9	4	2	1	3	5.4%
Rectal fistula	4	7.1%	0	0	2	2	4	7.1%
Rectal pain	4	7.1%	1	2	1	N/A	1	1.8%
Diarrhea	4	7.1%	0	4	0	0	0	0.0%
Proctitis	4	7.1%	1	3	0	0	0	0.0%
Constipation	3	5.4%	3	0	0	0	0	0.0%
Colitis	1	1.8%	0	1	0	0	1	1.8%
Anal pain	1	1.8%	0	1	0	N/A	0	0.0%
Rectal ulcer	1	1.8%	0	1	0	0	0	0.0%
General toxicity								
Pelvic pain	7	12.5%	1	4	2	N/A	1	1.8%
Pelvic soft tissue necrosis	6	10.7%	N/A	1	3	2	5	8.9%
Sepsis	2	3.6%	N/A	N/A	0	2	2	3.6%
Skin ulceration	2	3.6%	0	0	2	0	1	1.8%
Abdominal Pain	2	3.6%	2	0	0	N/A	0	0.0%
Fatigue	1	1.8%	1	0	0	N/A	0	0.0%
Subcutaneous emphysema	1	1.8%	0	1	0	N/A	0	0.0%
Weight loss	1	1.8%	0	0	1	0	0	0.0%

AE—adverse effect; SAE—serious adverse effect; N/A—not applicable; Explanation: Six cases (10.7%) of pelvic soft tissue necrosis mean that the AE was observed in six separate patients, but each of these patients could have experienced the AE multiple times. Only the highest grade of each distinct AE per patient is reported in this table. A complete list of all the events can be found in Supplementary File S1.

## Data Availability

Majority of the data is presented in Supplementary File S1. Specific anonymized data can be shared by the authors upon a reasonable request.

## Supplementary Materials

The following supporting information can be downloaded at https://www.mdpi.com/article/10.3390/cancers15041180/s1, Supplementary File S1: Case-by-case list of adverse events found in each individual patient included in the study; Supplementary File S2: Supplementary Tables S1–S6, including uni- and multivariate Cox regression models for the occurrence of grade ≥ 3 toxicity, LC, FFDM, PFS, BC and OS; Supplementary File S3: PFS and BC. Table S1: Cox Regression model analysing the association between clinicopathologic features and severe toxicity (grade ≥ 3) in 56 patients treated with hypofractionated salvage re-irradiation for local post-radiotherapy prostate cancer recurrence; Table S2: Cox Regression model analysing the association between clinicopathologic features and local control in 56 patients treated with hypofractionated salvage re-irradiation for local post-radiotherapy prostate cancer recurrence; Table S3: Cox Regression model analysing the association between clinicopathologic features and freedom from distant metastases in 56 patients treated with hypofractionated salvage re-irradiation for local post-radiotherapy prostate cancer recurrence; Table S4: Cox Regression model analysing the association between clinicopathologic features and progression-free survival in 56 patients treated with hypofractionated salvage re-irradiation for local post-radiotherapy prostate cancer recurrence; Table S5: Cox Regression model analysing the association between clinicopathologic features and biochemical control in 56 patients treated with hypofractionated salvage re-irradiation for local post-radiotherapy prostate cancer recurrence; Table S6: Cox Regression model analysing the association between clinicopathologic features and overall survival in 56 patients treated with hypofractionated salvage re-irradiation for local post-radiotherapy prostate cancer recurrence.
