# Supplementary material for: Long-Term Outcomes of Stereotactic Body Radiotherapy (SBRT) for Intraprostatic Relapse after Definitive Radiotherapy for Prostate Cancer: Patterns of Failure and Association between Volume of Irradiation and Late Toxicity
Source: Cancers (Basel). 2023 Feb 13;15(4):1180. doi: 10.3390/cancers15041180 (PMC9954604; doi:10.3390/cancers15041180)
Supplement: Supplementary file 1 [file cancers-15-01180-s001.zip › Supplementary File S2 supplementary tables.pdf]

Supplementary Table S1. Cox Regression model analysing the association between clinicopathologic features and severe toxicity (grade ≥3) in 56 patients treated with hypofractionated salvage re-irradiation for local post-radiotherapy prostate cancer recurrence.

	Univariable analysis		Multivariate analysis	
Variable:	HR (95% CI)	p-value	HR (95% CI)	p-value
Age [years]	1 (0.94-1.06)	0.888		
PSA max [ng/mL] (1st RT)	1 (0.99-1.01)	0.788		
ISUP 2-3 vs 1 (1st RT)	1.64 (0.59-4.52)	0.341		
ISUP 4-5 vs 1 (1st RT)	0.45 (0.1-2.06)	0.305		
T2b-c vs T1c-T2a (1st RT)	0.65 (0.22-1.87)	0.422		
T3a-b vs T1c-T2a (1st RT)	0.38 (0.07-1.96)	0.248		
ADT - yes vs no (1st RT)	1.37 (0.51-3.68)	0.535		
BT vs EBRT (1st RT)	1.16 (0.33-3.99)	0.819		
PSA nadir (1st RT) [ng/mL]	0.93 (0.5-1.76)	0.834		
Time from 1st RT to sSBRT [months]	1 (0.99-1.01)	0.578		
ADT - yes vs no (sSBRT)	0.32 (0.12-0.84)	0.02	0.35 (0.13-0.93)	0.035
ADT duration (months)	0.99 (0.98-1.01)	0.265		
CRPC at sSBRT (yes vs no)	0.79 (0.26-2.41)	0.674		
Oligometastases at sSBRT (yes vs no)	1.38 (0.45-4.19)	0.574		
PSA max at sSBRT [ng/mL]	0.93 (0.82-1.04)	0.212		
RT dose ($BED_{1.5}$) (sSBRT)	1 (0.99-1.02)	0.941		
RT dose (BED_{10}) (sSBRT)	1.02 (0.95-1.09)	0.623		
RT dose ($BED_{1.5}$) (sSBRT + 1st RT combined)	1 (0.99-1.01)	0.849		
RT dose (BED_{10}) (sSBRT + 1st RT combined)	1.03 (0.96-1.1)	0.4		
Focal vs. whole gland sSBRT	0.11 (0.01-0.83)	0.032		
PTV [cc] (sSBRT)	1.01 (1-1.03)	0.013	1.01 (1-1.02)	0.025

1st RT – refers to primary treatment (i.e. maximum PSA before first radiotherapy); sSBRT – refers to salvage re-irradiation; BT includes both BT boost, HDR and LDR BT; co-varieties significant at a p-value of <0.05 are highlighted in red.

Supplementary Table 2. Cox Regression model analysing the association between clinicopathologic features and local control in 56 patients treated with hypofractionated salvage re-irradiation for local post-radiotherapy prostate cancer recurrence.

	Univariate analysis		Multivariate analysis	
Variable:	HR (95% CI)	p-value	HR (95% CI)	p-value
Age [years]	1.04 (0.97-1.12)	0.237		
PSA max [ng/mL] (1st RT)	1.01 (1-1.01)	0.028	1.01 (1-1.01)	0.021
ISUP 2-3 vs 1 (1st RT)	1.16 (0.28-4.74)	0.838	1.15 (0.22-5.99)	0.865
ISUP 4-5 vs 1 (1st RT)	2.78 (0.91-8.48)	0.072	4.86 (1.06-22.36)	0.042
T2b-c vs T1c-T2a (1st RT)	4.6 (1.47-14.38)	0.009	11.34 (2.43-52.87)	0.001
T3a-b vs T1c-T2a (1st RT)	1.89 (0.47-7.57)	0.371	2.26 (0.46-11.05)	0.002
ADT - yes vs no (1st RT)	0.62 (0.22-1.72)	0.362		
BT vs EBRT (1st RT)	1.01 (0.23-4.49)	0.99		
PSA nadir (1st RT) [ng/mL]	0.69 (0.32-1.5)	0.349		
Time from 1st RT to sSBRT [months]	1 (0.99-1.01)	0.411		
ADT - yes vs no (sSBRT)	0.41 (0.14-1.15)	0.09	0.3 (0.08-1.08)	0.066
ADT duration (months)	1 (0.99-1.02)	0.605		
CRPC at sSBRT (yes vs no)	1.25 (0.43-3.65)	0.681		
Oligometastases at sSBRT (yes vs no)	1.11 (0.31-3.96)	0.88		
PSA max at sSBRT [ng/mL]	1.02 (0.98-1.05)	0.284		
RT dose ($BED_{1.5}$) (sSBRT)	1 (0.98-1.01)	0.676		
RT dose ($BED_{1.5}$) (sSBRT + 1st RT combined)	1 (0.98-1.01)	0.339		
Focal vs. whole gland sSBRT	0.86 (0.3-2.45)	0.782		
PTV [cc] (sSBRT)	1 (0.99-1.02)	0.753		

1st RT – refers to primary treatment (i.e. maximum PSA before first radiotherapy); sSBRT – refers to salvage re-irradiation; BT includes both BT boost, HDR and LDR BT; co-varieties significant at a p-value of <0.05 are highlighted in red.

Supplementary Table 3. Cox Regression model analysing the association between clinicopathologic features and freedom from distant metastases in 56 patients treated with hypofractionated salvage re-irradiation for local post-radiotherapy prostate cancer recurrence.

	Univariate analysis		Multivariate analysis	
Variable:	HR (95% CI)	p-value	HR (95% CI)	p-value
Age [years]	1.04 (0.98-1.11)	0.209		
PSA max [ng/mL] (1st RT)	1 (1-1.01)	0.4		
ISUP 2-3 vs 1 (1st RT)	2.09 (0.63-7)	0.263	1.16 (0.29-4.74)	0.833
ISUP 4-5 vs 1 (1st RT)	4.81 (1.59-14.56)	0.005	5.01 (1.27-19.76)	0.021
T2b-c vs T1c-T2a (1st RT)	0.24 (0.72-6.93)	0.161	3.38 (0.84-13.55)	0.085
T3a-b vs T1c-T2a (1st RT)	3 (1.03-8.73)	0.044	1.98 (0.28-13.88)	0.493
ADT - yes vs no (1st RT)	2.14 (0.8-5.69)	0.127	2.5 (0.83-7.59)	0.105
BT vs EBRT (1st RT)	0.29 (0.04-2.17)	0.228		
PSA nadir (1st RT) [ng/mL]	0.63 (0.28-1.41)	0.265		
Time from 1st RT to sSBRT [months]	1 (0.99-1.01)	0.461		
ADT - yes vs no (sSBRT)	0.94 (0.3-2.92)	0.913		
ADT duration (months)	1.01 (1-1.02)	0.029	1 (0.99-1.02)	0.622
CRPC at sSBRT (yes vs no)	1.79 (0.7-4.6)	0.223		
Oligometastases at sSBRT (yes vs no)	1.64 (0.53-5.06)	0.392		
PSA max at sSBRT [ng/mL]^	1.02 (0.99-1.05)	0.138	1.01 (0.98-1.04)	0.596
PET used in treatment planning (yes vs no)	0.78 (0.29-2.05)	0.61		
RT dose ($BED_{1.5}$) (sSBRT)	1 (0.99-1.01)	0.65		
RT dose ($BED_{1.5}$) (sSBRT + 1st RT combined)	1 (0.99-1.01)	0.53		
Focal vs. whole gland sSBRT	0.68 (0.25-1.86)	0.451		
PTV [cc] (sSBRT)	1 (0.99-1.02)	0.95		

1st RT – refers to primary treatment (i.e. maximum PSA before first radiotherapy); sSBRT – refers to salvage re-irradiation; BT includes both BT boost, HDR and LDR BT; co-varieties significant at a p-value of <0.05 are highlighted in red.

Supplementary Table 4. Cox Regression model analysing the association between clinicopathologic features and progression-free survival in 56 patients treated with hypofractionated salvage re-irradiation for local post-radiotherapy prostate cancer recurrence.

	Univariate analysis		Multivariate analysis	
Variable:	HR (95% CI)	p-value	HR (95% CI)	p-value
Age [years]	1.03 (0.98-1.07)	0.213		
PSA max [ng/mL] (1st RT)	1 (1-1.01)	0.227		
ISUP 2-3 vs 1 (1st RT)	1.73 (0.7-4.26)	0.233	1.29 (0.5-3.31)	0.602
ISUP 4-5 vs 1 (1st RT)	3.48 (1.62-7.48)	0.001	4.15 (1.73-9.94)	0.001
T2b-c vs T1c-T2a (1st RT)	2.6 (1.17-5.76)	0.019	2.89 (1.13-7.36)	0.026
T3a-b vs T1c-T2a (1st RT)	2.39 (1-5.71)	0.05	0.8 (0.21-3.06)	0.74
ADT - yes vs no (1st RT)	1.48 (0.74-2.96)	0.262		
BT vs EBRT (1st RT)	0.82 (0.32-2.12)	0.685		
PSA nadir (1st RT) [ng/mL]	0.88 (0.56-1.36)	0.556		
Time from 1st RT to sSBRT [months]	1 (0.99-1.01)	0.738		
ADT - yes vs no (sSBRT)	0.83 (0.39-1.77)	0.623		
ADT duration (months)	1.01 (1-1.02)	0.007	1.01 (1-1.02)	0.162
CRPC at sSBRT (yes vs no)	1.65 (0.79-3.45)	0.181		
Oligometastases at sSBRT (yes vs no)	1.05 (0.46-2.42)	0.91		
PSA max at sSBRT [ng/mL]	0.97 (0.48-1.95)	0.45		
PET used in treatment planning (yes vs no)	0.76 (0.36-1.63)	0.484		
RT dose ($BED_{1.5}$) (sSBRT)	1 (0.99-1.01)	0.793		
RT dose ($BED_{1.5}$) (sSBRT + 1st RT combined)	1 (0.99-1)	0.35		
Focal vs. whole gland sSBRT	0.93 (0.44-1.95)	0.848		
PTV [cc] (sSBRT)	1 (0.99-1.01)	0.98		

1st RT – refers to primary treatment (i.e. maximum PSA before first radiotherapy); sSBRT – refers to salvage re-irradiation; BT includes both BT boost, HDR and LDR BT; co-varieties significant at a p-value of <0.05 are highlighted in red.

Supplementary Table 5. Cox Regression model analysing the association between clinicopathologic features and biochemical control in 56 patients treated with hypofractionated salvage re-irradiation for local post-radiotherapy prostate cancer recurrence.

	Univariate analysis		Multivariate analysis	
Variable:	HR (95% CI)	p-value	HR (95% CI)	p-value
Age [years]	1.03 (0.97-1.1)	0.306		
PSA max [ng/mL] (1st RT)	1 (1-1.01)	0.663		
ISUP 2-3 vs 1 (1st RT)	2.36 (0.62-8.97)	0.209	1.95 (0.44-8.66)	0.378
ISUP 4-5 vs 1 (1st RT)	5.38 (1.83-15.87)	0.002	8.85 (2.16-36.22)	0.002
T2b-c vs T1c-T2a (1st RT)	2.49 (0.83-7.48)	0.104	3.69 (0.77-17.71)	0.103
T3a-b vs T1c-T2a (1st RT)	2.08 (0.68-6.39)	0.202	0.37 (0.04-3.04)	0.354
ADT - yes vs no (1st RT)	2.05 (0.77-5.44)	0.151		
BT vs EBRT (1st RT)	1.03 (0.23-4.6)	0.969		
PSA nadir (1st RT) [ng/mL]	0.76 (0.38-1.52)	0.435		
Time from 1st RT to sSBRT [months]	1 (0.99-1.01)	0.431		
ADT - yes vs no (sSBRT)	1.02 (0.33-3.17)	0.975		
ADT duration (months)	1.01 (1-1.02)	0.008	1.01 (0.99-1.04)	0.387
CRPC at sSBRT (yes vs no)	2.52 (1-6.34)	0.05	2.45 (0.5-11.93)	0.267
Oligometastases at sSBRT (yes vs no)	1.13 (0.37-3.5)	0.828		
PSA max at sSBRT [ng/mL]	1.03 (1-1.07)	0.026	1.01 (0.97-1.05)	0.613
PET used in treatment planning (yes vs no)	1.02 (0.36-2.88)	0.964		
RT dose ($BED_{1.5}$) (sSBRT)	1 (0.98-1.01)	0.77		
RT dose ($BED_{1.5}$) (sSBRT + 1st RT combined)	0.99 (0.98-1.01)	0.316		
Focal vs. whole gland sSBRT	0.76 (0.29-2.03)	0.589		
PTV [cc] (sSBRT)	1 (0.98-1.01)	0.94		

1st RT – refers to primary treatment (i.e. maximum PSA before first radiotherapy); sSBRT – refers to salvage re-irradiation; BT includes both BT boost, HDR and LDR BT; co-varieties significant at a p-value of <0.05 are highlighted in red.

Supplementary Table 4. Cox Regression model analysing the association between clinicopathologic features and overall survival in 56 patients treated with hypofractionated salvage re-irradiation for local post-radiotherapy prostate cancer recurrence.

	Univariate analysis		Multivariate analysis	
Variable:	HR (95% CI)	p-value	HR (95% CI)	p-value
Age [years]	1.03 (0.98-1.09)	0.219		
PSA max [ng/mL] (1st RT)	1 (0.99-1.01)	0.82		
ISUP 2-3 vs 1 (1st RT)	1.07 (0.35-3.28)	0.907		
ISUP 4-5 vs 1 (1st RT)	1.9 (0.78-4.6)	0.156		
T2b-c vs T1c-T2a (1st RT)	2.02 (0.84-4.83)	0.116	2.32 (0.95-5.68)	0.065
T3a-b vs T1c-T2a (1st RT)	0.9 (0.26-3.12)	0.863	0.99 (0.28-3.5)	0.992
ADT - yes vs no (1st RT)	2.21 (0.91-5.33)	0.078		
BT vs EBRT (1st RT)	0.79 (0.24-2.66)	0.709		
PSA nadir (1st RT) [ng/mL]	0.96 (0.56-1.62)	0.868		
Time from 1st RT to sSBRT [months]	1 (0.99-1.01)	0.75		
ADT - yes vs no (sSBRT)	0.75 (0.31-1.81)	0.523		
ADT duration (months)	1 (0.99-1.01)	0.482		
CRPC at sSBRT (yes vs no)	0.96 (0.38-2.41)	0.93		
Oligometastases at sSBRT (yes vs no)	1.05 (0.4-2.81)	0.916		
PSA max at sSBRT [ng/mL]	1.01 (0.98-1.04)	0.479		
PET used in treatment planning (yes vs no)	0.62 (0.27-1.45)	0.274		
RT dose ($BED_{1.5}$) (sSBRT)	1 (0.99-1.02)	0.513		
RT dose ($BED_{1.5}$) (sSBRT + 1st RT combined)	1 (0.99-1.01)	0.887		
Focal vs. whole gland sSBRT	0.62 (0.25-1.51)	0.292		
PTV [cc] (sSBRT)	1.01 (1.01-1.03)	0.029	1.01 (1-1.03)	0.019

1st RT – refers to primary treatment (i.e. maximum PSA before first radiotherapy); sSBRT – refers to salvage re-irradiation; BT includes both BT boost, HDR and LDR BT; co-varieties significant at a p-value of <0.05 are highlighted in red.
