# Supplementary material for: Long-Term Outcomes of Stereotactic Body Radiotherapy (SBRT) for Intraprostatic Relapse after Definitive Radiotherapy for Prostate Cancer: Patterns of Failure and Association between Volume of Irradiation and Late Toxicity
Source: Cancers (Basel). 2023 Feb 13;15(4):1180. doi: 10.3390/cancers15041180 (PMC9954604; doi:10.3390/cancers15041180)
Supplement: Supplementary file 1 [file cancers-15-01180-s001.zip › Supplementary-File-S3.pdf]

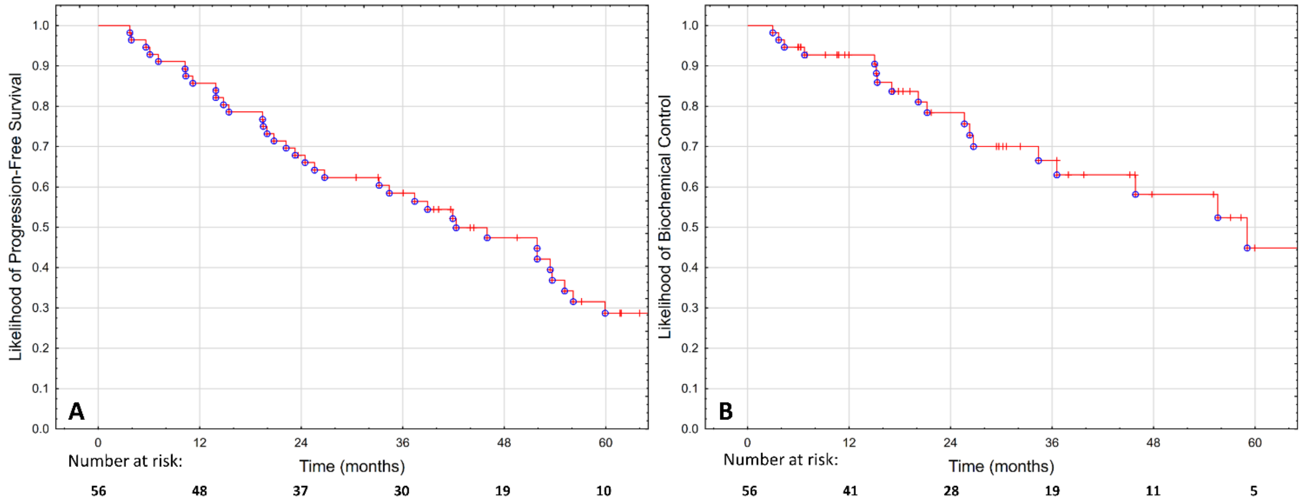

Supplementary Figure S1. Progression-Free Survival (**A**) and Biochemical Control (**B**) in 56 patients treated with hypofractionated salvage re-irradiation for local post-radiotherapy prostate cancer recurrence.

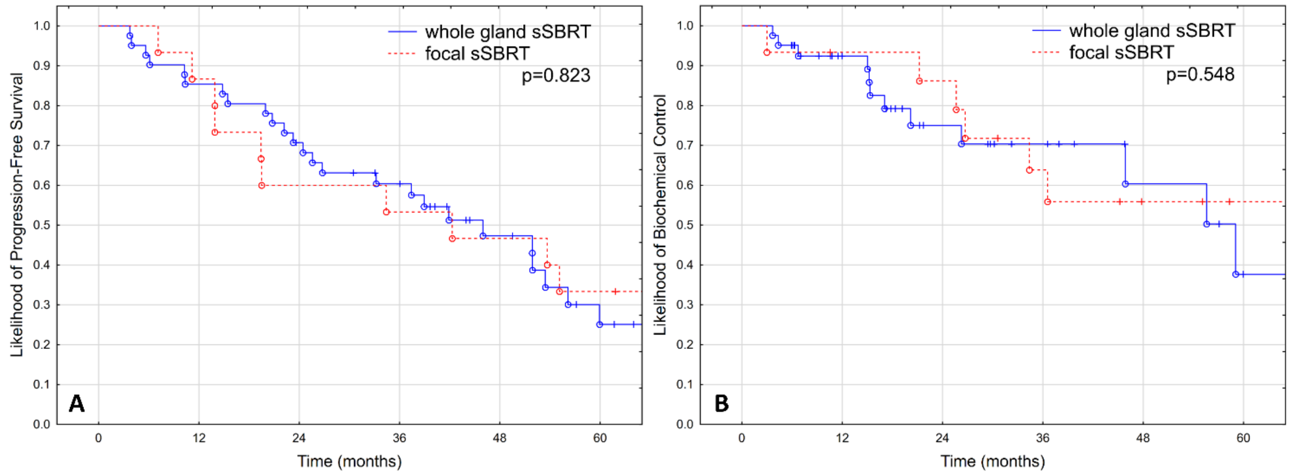

Supplementary Figure S2. Supplementary Figure 2. Progression-Free Survival (A) and Biochemical Control (B) depending on the extent of irradiation in patients receiving hypofractionated salvage re-irradiation for local post-radiotherapy prostate cancer recurrence.
